# CHRNB2 represses pancreatic cancer migration and invasion via inhibiting β-catenin pathway

**DOI:** 10.1186/s12935-022-02768-8

**Published:** 2022-11-07

**Authors:** Cheng Qin, Tianhao Li, Yuanyang Wang, Bangbo Zhao, Zeru Li, Tianyu Li, Xiaoying Yang, Yutong Zhao, Weibin Wang

**Affiliations:** 1 Department of General Surgery, State Key Laboratory of Complex Severe and Rare Diseases, Peking Union Medical College Hospital, Chinese Academy of Medical Sciences and Peking Union Medical College, Beijing, China; 2 Division of Plastic Surgery, Department of Surgery, Peking Union Medical College Hospital, Chinese Academy of Medical Sciences and Peking Union Medical College, Beijing, China

**Keywords:** Pancreatic cancer, Lymph node ratio, Weighted gene co-expression network analysis, CHRNB2, β-catenin pathway

## Abstract

**Background:**

Pancreatic cancer is one of the most lethal disease with highly fatal and aggressive properties. Lymph node ratio (LNR), the ratio of the number of metastatic lymph nodes to the total number of examined lymph nodes, is an important index to assess lymphatic metastasis and predict prognosis, but the molecular mechanism underlying high LNR was unclear.

**Methods:**

Gene expression and clinical information data of pancreatic cancer were obtained from The Cancer Genome Atlas (TCGA) and Gene Expression Omnibus (GEO). Patients in TCGA were averagely divided into low and high LNR groups. Then, Weighted Gene Co-expression Network Analysis (WGCNA) was utilized to build co-expression network to explore LNR-related modules and hub genes. GO and KEGG analysis was performed to find key pathways related to lymph node metastasis. Next, GSE101448 and the overall survival data in TCGA was employed to further select significant genes from hub genes. Considering the key role of CHRNB2 in LNR and survival, gene set enrichment analysis (GSEA) was applied to find pathways related to CHRNB2 expression in pancreatic cancer. The contribution of CHRNB2 to migrative and invasive ability of pancreatic cancer cells was confirmed by Transwell assays. We finally explored the role of CHRNB2 in EMT and β-catenin pathway via Western Blot.

**Results:**

High LNR was significantly related to high T stages and poor prognosis. In WGCNA, 14 hub genes (COL5A1, FN1, THBS2, etc.) were positively related to high LNR, 104 hub genes (FFAR1, SCG5, TMEM63C, etc.) were negatively related to high LNR. After taking the intersection with GSE101448, 13 genes (CDK5R2, SYT7, CACNA2D2, etc.) which might prevent lymph node metastasis were further selected. Among them, CHRNB2 showed the strongest relationship with long survival. Moreover, CHRNB2 also negatively related to the T stages and LNR. Next, knockdown of CHRNB2 expression could acetylcholine (ACh)-independently increase the migration and invasion of pancreatic cancer cells, while CHRNB2 overexpression ACh-independently decrease the migration and invasion of pancreatic cancer cells. For exploring the underlying mechanism, CHRNB2 downregulated β-catenin pathway might through controlling its upstream regulators such as SOX6, SRY, SOX17, and TCF7L2.

**Conclusions:**

CHRNB2 negatively relates to lymph node metastasis in pancreatic cancer patients. CHRNB2 could inhibit β-catenin pathway, EMT, migration and invasion of pancreatic cancer cells via ACh-independent mechanism.

**Supplementary Information:**

The online version contains supplementary material available at 10.1186/s12935-022-02768-8.

## Introduction

Pancreatic cancer is one of the most lethal malignant tumors worldwide with a 5-year overall survival rate of 11% [1]. At present, radical operation combined with chemotherapy is the only way to cure pancreatic cancer. Because of early metastasis, about 80% of the patients have lost the opportunity for receiving radical surgery when diagnosed, which resulted in the high mortality of pancreatic cancer [2].

Both bloodborne and lymphatic metastasis are two primary pathways contributing to the distant metastasis of pancreatic cancer [3]. Due to the tumor-draining lymph nodes would be cleaned during operation and examined by postoperative pathology, it provided an objective index for assessing and predicting distant metastasis [4]. Besides that, recent reports also suggested that lymph node metastasis could induce immune suppression in pancreatic cancer [5]. Thus, understanding the inner regulatory mechanism of lymphatic metastasis is of vital importance for not only predicting distant metastasis and prognosis but also restraining the progression of pancreatic cancer.

N staging is a routine index that reflects the status of lymphatic metastasis. Previous clinical studies and guidelines suggested that the minimal number of examined lymph nodes should be more than 11 [6]. However, it is impossible to harvest enough lymph nodes for all patients in clinical practice. Due to inadequate examined lymph nodes, lymph node ratio (LNR), determined by dividing the total number of metastatic lymph nodes by the total number of examined lymph nodes, is proved to be another effective index to assess lymphatic metastasis [7, 8]. Previous study has shown that pancreatic cancer patients with LNR > 0.2 had significantly lower survival time [9]. Moreover, it exhibited superior role than N staging in predicting overall survivals when the number of examined lymph node was less than 15 [10]. Therefore, LNR provided an effective way to assess lymph node metastasis regardless of examined lymph nodes number.

In order to identify the hub-genes that associate with lymph node metastasis of patients with pancreatic cancer, we employed The Cancer Genome Atlas (TCGA) database and performed a Weighted Gene Co-expression Network Analysis (WCGNA) algorithm, a biological algorithm used to study intrinsic features of gene sets and find relationships between phenotypes and gene modules [11]. WGCNA was increasingly applied in exploring the association between phenotypes and special gene expression in cancer. In recent years, several key genes have been revealed by WGCNA. DACT3, TNS1, and MSRB3 were suggested to promote the lymph node metastasis of bladder cancer [12]. For colorectal cancer, FSTL3 could accelerate lymph node metastasis and inhibitory immune microenvironment formation [13].However, the underlying mechanism between those genes and lymph node metastasis is unclear. Moreover, there are still lack of relevant studies in pancreatic cancer. After constructing gene modules relating to LNR, we screened out several hub genes, among which Cholinergic receptor nicotinic beta 2 subunit (CHRNB2) was chosen to be further investigated. Gene Set Enrichment Analysis (GSEA) was then applied to find the negative relationship between CHRNB2 expression and Wnt/β-catenin pathway [14].

CHRNB2 is an essential part of the nicotinic acetylcholine receptor (nAChR). Previous studies found CHRNB2 is related to nicotine addiction and epilepsy [15, 16]. But there is little research on CHRNB2 in cancer. The role of CHRNB2 in pancreatic cancer progression was not investigated before. Wnt/β-catenin pathway is vital for promoting epithelial–mesenchymal transition (EMT) and metastasis in pancreatic cancer [17]. The relationship between CHRNB2 and Wnt/β-catenin pathway was unclear. We therefore explored the function of CHRNB2 in pancreatic cancer and its role in Wnt/β-catenin pathway.

## Materials and methods

### Data acquisition

We obtained The Cancer Genome Atlas (TCGA) dataset from UCSC XENA website (https://xenabrowser.net/datapages/; up to March 1, 2022), which contained 179 pancreatic tumor tissues. After matching gene expression data and clinical information, 174 cases with complete LNR information and corresponding RNA sequencing data were finally included in this study. In addition, lymph node metastasis information and gene expression profiles of 24 primary pancreatic tumors of GSE101448, 36 paired normal-tumor samples of pancreatic cancer patients with gene expression profiles of GSE15471 were downloaded from the Gene Expression Omnibus (GEO, https://www.ncbi.nlm.nih.gov/geo/) [18]. The expression level of each gene was calculated as Transcripts Per Kilobase Million (TPM) with log2 transformation.

### Weighted gene co-expression network analysis (WGCNA)

GeneS expression data of TCGA dataset with high standard deviation (top 5000) were included for constructing co-expression network by ‘WGCNA’ package in R [11]. Briefly, outlier samples were ruled out by sample clustering. Mean connectivity and scale independence analysis were utilized to determine a soft threshold. Then, adjacency matrix of expressed genes was built and transformed to topological overlap matrix (TOM). Therefore, under the parameters including 0.25 for merged cut height and 30 for minimum module size, multiple genes with similar expression profiles could be attributed into one gene module. Pearson’s correlation analysis was performed to identify significant gene modules associated with high/low LNR.

### Hub genes detection and functional enrichment

The intrinsic connectivity of all genes in blue or green module was generated from WGCNA, and then the network was built in Cytoscape [19]. Molecular Complex Detection (MCODE) in Cytoscape was utilized to find hub genes which have the highest connectivity in the network [20]. We then employed DAVID database 6.8 to conduct Gene Ontology (GO) enrichment and the Kyoto Encyclopedia of Genes and Genomes (KEGG) pathway analysis for the hub genes in each statistically significant module (https://david-d.ncifcrf.gov/; up to March 1, 2022) [21, 22]. GSEA database was utilized to assess the correlation between CHRNB2 expression and Wnt/β-catenin pathway (http://www.gsea-msigdb.org/; up to March 1, 2022) [23].

### Cell culture

HPNE, PANC1, PATU8902, Mia-Paca-2, BxPC3, AsPC1 and T3M4 cell lines were bought from the American Type Culture Collection (Virginia, USA). Cell lines were cultured in RPMI1640 or DMEM (Gibco, USA) with 10% of fetal bovine serum (FBS) (ScienCell, USA) under the temperature of 37 °C with 5% carbon dioxide. 1% antibiotics was added in the medium.

### Constructs and cell transfection

The full-length CHRNB2 cDNA was subcloned into the empty vector (pcDNA 3.1) to generate CHRNB2 overexpression plasmid. Small interfering RNA targeting CHRNB2 (siCHRNB2-1 sense: 5′-GUGGACAUCACGUAUGACUTT-3′; siCHRNB2-1 antisense: 5′-AGUCAUACGUGAUGUCCACTT-3′; siCHRNB2-2 sense: 5′- CAGAUGUGGUCCUGUACAATT-3′; siCHRNB2-2 antisense: 5′-UUGUACAGGACCACAUCUGTT-3′) and negative control (NC) were purchased from Tsingke Co., Ltd. When the seeded cells in 6 well plates reached 60% confluence, we used Lipofectamine 3000 Reagent (ThermoFisher, USA) and OPTI-MEM (Invitrogen, USA) to transfect siRNA or plasmid into cells according to the instructions. The transfected cells were harvested to do downstream experiments after cultivation for 48 h.

### Cell migration and invasion experiment

For migration experiment, 3 × 10^4^ transfected cells were seeded into the Transwell chamber (Corning, USA) with FBS-free medium and the Transwell chambers were placed in 24 well plates with 10% FBS medium. After incubation for 24 h, the cells were fixed by methanol and stained by hematoxylin–eosin staining. For invasion experiment, 70 μL Matrigel matrix (Corning, USA) was added before seeding of cells. 5 random fields were utilized to count cell number. Statistical findings are identified by P-value < 0.05.

### Western blot

Western blot was performed as previously described [24]. The primary antibodies used were: anti-CHRNB2 (Abcam, ab41174) for Fig. 5, anti-CHRNB2 (Santa Cruz, sc-58596) for other figures, anti-N-Cadherin (Proteintech, 22018–1-AP), anti-Vimentin (Proteintech, 10366–1-AP), anti-β-catenin (CST, 8480), anti-Cyclin D1 (Abcam, AB16663), anti-CD44 (Proteintech, 15675–1-AP), anti-C-Myc (CST, 5605), anti-SNAIL1(Proteintech, 13099–1-AP), anti-SNAIL2 (Proteintech, 12129–1-AP), anti-Vinculin (Proteintech, 66305–1-Ig) and anti-GAPDH (Proteintech, 10494–1-AP). Quantitative analysis of Western Blot was performed by ImageJ software. Fold changes under individual blots represented the ratio to relevant control (numbers in *italic* font).

### RNA isolation and qRT-PCR

The pancreatic cancer and paracancer tissues with corresponding clinical and pathologic data of 6 patients who underwent pancreatic cancer surgery in the Peking Union Medical College Hospital in 2021 were collected. All the patients enrolled in this study had written the informed consent. The Ethics Committee of Peking Union Medical College Hospital has approved this project. TRizol reagent (Invitrogen) was used for the extracting total RNA from tissue and pancreatic cancer cell lines. cDNA synthesis was performed using the First Strand cDNA Synthesis Kit (Thermo Scientific) following the manufacturer's instructions. Quantitative PCR was conducted using SYBR Green Mix (Applied Biosystems) based on the manufacturer’s instructions as well. The chrnb2 forward primer used was CTTGTCACCTTCTCCATCGTC, and the chrnb2 reverse primer used was TTCTCCAGGAAGACGACCTT. β-actin forward: CTCCATCCTGGCCTCGCT GT; β-actin reverse: GCTGTCACCTTCACCGTTCC; cd44 forward: TCTGAATCAGATGGACACTCAC; cd44 reverse: CATTGCCACTGTTGATCACTAG; c-myc forward: CGACGAGACCTTCATCAAAAAC; c-myc reverse: CTTCTCTGAGACGAGCTTGG; cyclin d1 forward: GTCCTACTTCAAATGTGTGCAG; cyclin d1 reverse: GGGATGGTCTCCTTCATCTTAG; sry forward: GCGAAGATGCTGCCGAAGAATTG; sry reverse: AGTGTGTGGCTTTCGTACAGTCATC; sox6 forward: CAACTACCACACCATCGCCTCAG; sox6 reverse: GCTTCCGCCATCTGTCTTCATACC; sox17 forward: TCGGGGACATGAAGGTGAAGGG; sox17 reverse: CGTCCTTAGCCCACACCATGAAAG; tcf7l2 forward: CCACAGCTCTGACCGTCAATGC; tcf7l2 reverse: CCCGTCGTGTGTAGCGTATGATG.

### Cell cycle analysis

48 h after transfection, transfected cells were fixed in precooled ethanol at 4 °C overnight. Propidium iodide, reaction buffer, and RNase A (beyotime, China) were utilized to determine DNA content. flow cytometry was performed by the Invitrogen (Attune Nxt 2L-BR). FlowJo software was utilized to analyze the cell cycle data derived from the flow cytometry.

### Statistical analysis

The WGCNA analysis and Kaplan–Meier analysis between high/low LNR patients was conducted by R software (v.3.8.0). The overall survival curves of genes were obtained from online analysis website GEPIA2 (http://gepia2.cancer-pku.cn/) [25]. Chi-square test was employed to calculate baseline clinical information. Unpaired T test was utilized to compare the gene expression and the number of migrated or invasive cells between different groups. Statistical analyses were performed by GraphPad Prism (v.8.1.2, USA). P < 0.05 was considered statically significant.

## Results

### High LNR related to high T stages and poor prognosis in pancreatic cancer

**Fig. 1:**
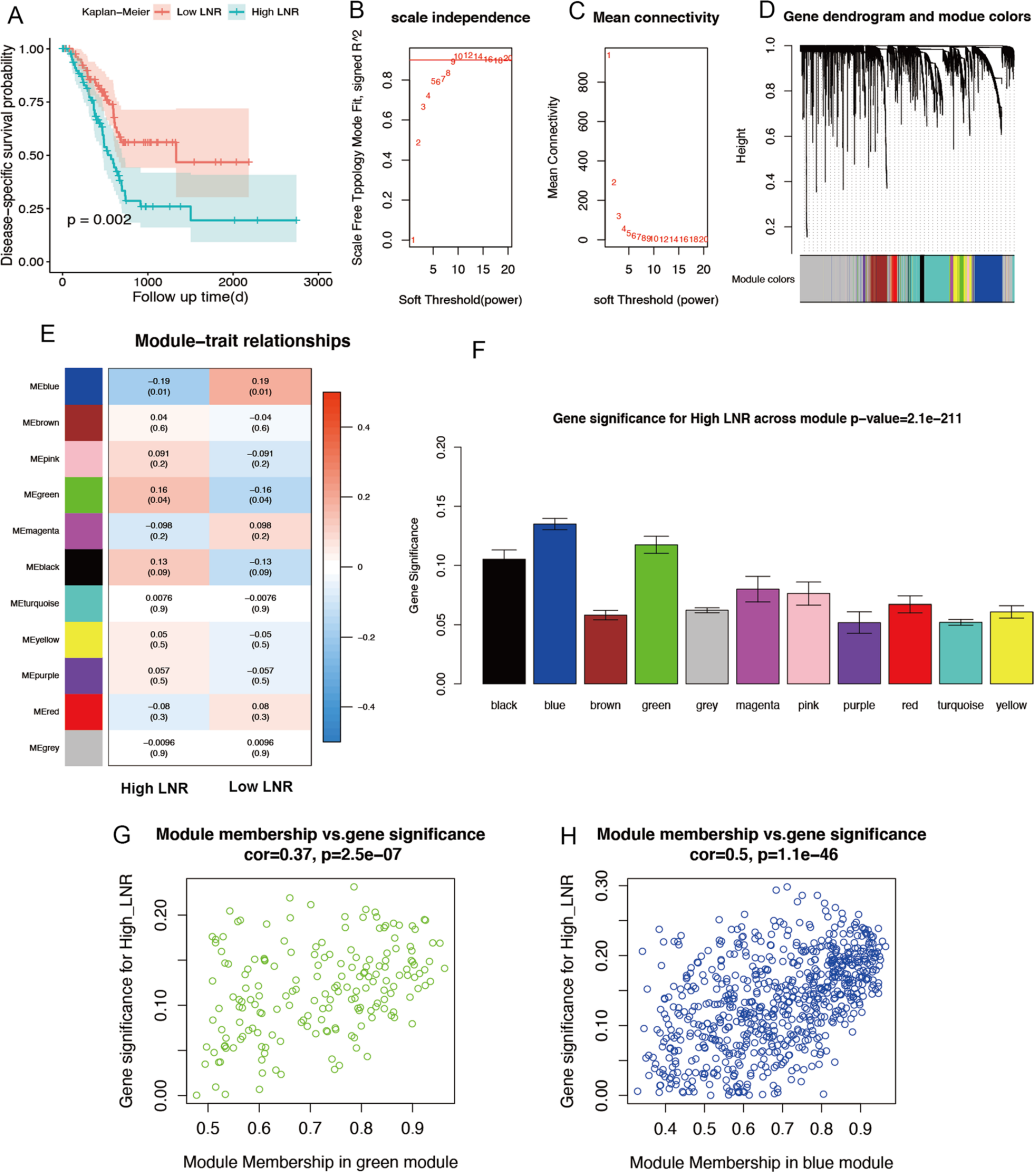
WGCNA analysis based on LNR in pancreatic cancer patients. **A** High LNR correlates with a poor prognosis in pancreatic cancer patients (P = 0.002). **B** Network topology analysis for soft-thresholding powers. X-axis indicates various soft-thresholding powers. Y-axis means the correlation coefficient between log [P(k)] and log (k). The red line represents correlation coefficient = 0.9. **C** Mean network connectivity under distinct soft-thresholding powers. **D** Clustering all included genes into modules based on the topological overlap, assigning them into different colors. **E** Module-trait associations. The number in box is correlation coefficient, the number in brackets represents corresponding P value. Red indicates high positive correlation, whereas blue means negative correlation. **F** Bar plots showed blue and green modules are two most key modules related to LNR. **G**, **H** Scatterplots exhibited the module membership of genes versus high LNR in green and blue modules

Firstly, we analyzed the clinical data of pancreatic cancer patients with high/low LNR in TCGA database. We found that the patients with high LNR had significantly poor prognosis (Fig. 1A). Besides the overall survival, the baseline clinical information between patients with high/low LNR was also different (Table 1). Patients with high LNR were preferred to have higher T stages, which supported the evidence that lymphatic metastasis regularly accompanied with tumor growth and progression. lymph node metastasis is known to be caused by the interaction between cancer cells and lymphatic endothelial cells [4]. The result emphasized the intrinsic role of pancreatic cancer cells in lymph node metastasis.

**Table 1:** Relationship between low/high LNR and clinical information in pancreatic cancer patients

Variables	LNR		Total	P value
	Low(n = 91)	High(n = 91)		
Sex				
Female	38	44	82	0.577
Male	52	48	100	
Age				
≤ 65	44	49	93	0.555
> 65	46	43	89	
T Stage				
T1&T2	22	9	31	0.009^**^
T2&T3	68	83	151	
M stage				
M0	37	47	84	0.062
M1	5	1	6	
Histological grade				
G1&G2	64	62	126	0.501
G3&G4	24	29	53	
Tumor location				
Head	62	74	136	0.073
Non-head	28	18	46	
Somker				
No	36	31	67	0.326
Yes	36	43	79	
Alcohol history				
No	36	29	65	0.291
Yes	46	52	98	
Diabetes history				
No	58	51	109	0.362
Yes	16	20	36	
Chronic pancreatitis history				
No	67	60	127	0.65
Yes	6	7	13	
Family history of cancer				
No	24	21	45	0.851
Yes	34	32	66	

** P < 0.01

### WGCNA Based on LNR and identification of key modules

Gene expression data of 174 pancreatic cancer cases with explicit LNR information were collected to build weight co-expression network by R package “WGCNA”. Soft threshold power β = 8 was selected to construct similarity of all gene pairs (Fig. 1B, C). Then, 13 gene modules with different colors were generated by dynamic tree cut algorithm (Fig. 1D). The association pattern among different modules were plotted as heatmaps (Fig. 1E). To find key modules and co-expressed genes which were significantly correlated with clinical features, we identified the relationship between LNR with different modules by statistical analysis. Among 13 gene modules, we found that green module was positively related to high LNR and blue module was negatively associated with high LNR (Fig. 1E). Moreover, compared with other modules, blue module and green modules possessed more gene significance for LNR (Fig. 1F). The correlation between gene significance and module membership in green and blue modules were illustrated respectively (Fig. 1G, H).

### Function analysis of key modules and identification of hub genes

**Fig. 2:**
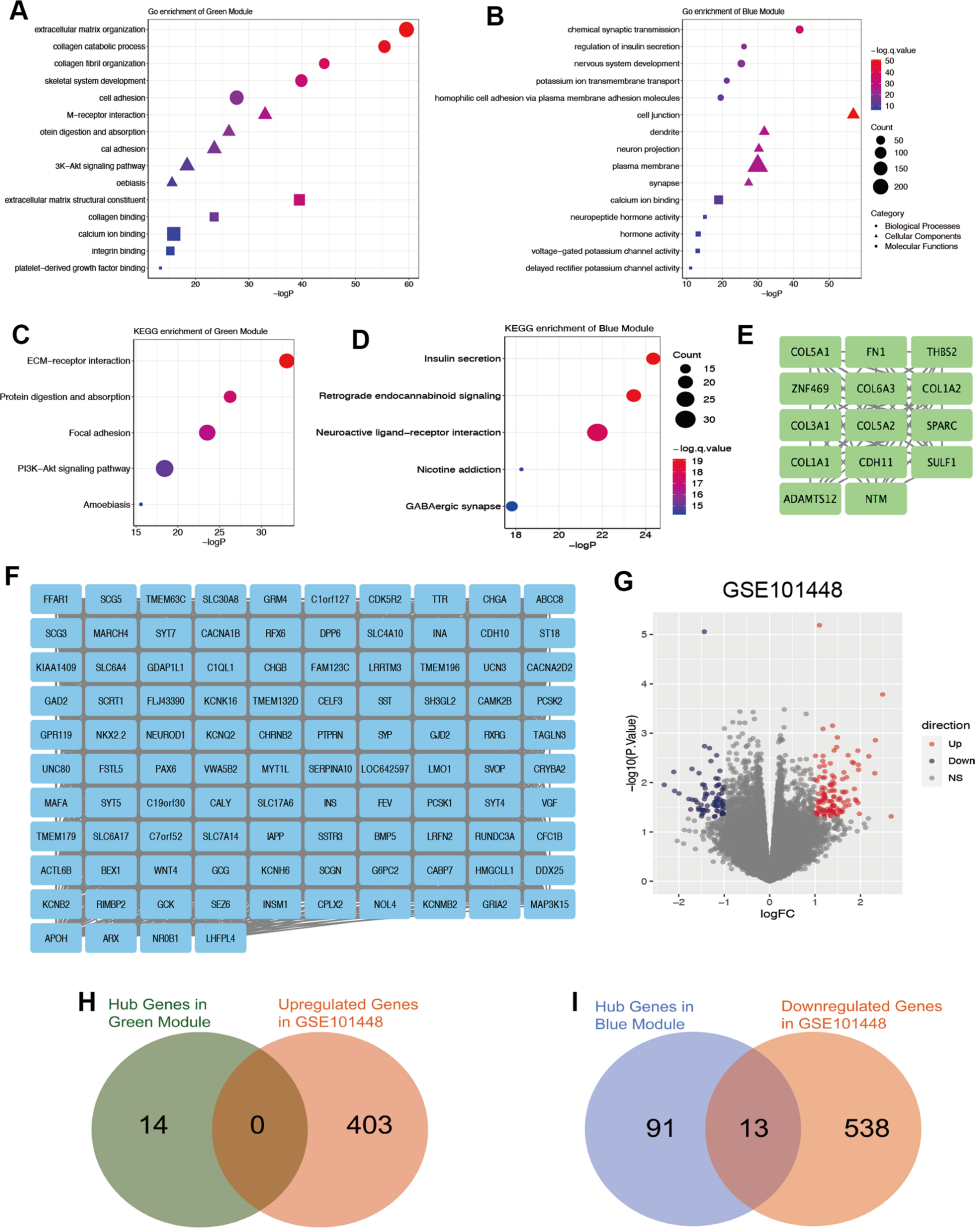
Hub genes in green and blue modules. **A** Go enrichment of green module. Top 5 significant pathways in different aspects. Circle represents biological process, triangle represents cellular component, rectangle represents molecular function. **B** Go enrichment of blue module. Top 5 significant pathways in different aspects. Circle represents biological process, triangle represents cellular component, rectangle represents molecular function. **C**, **D** KEGG enrichment of green and blue modules respectively. **E**, **F** Hub genes in green and blue modules respectively, which is calculated by the MCODE in Cytoscape. **G** Differential expressed genes in GSE10448. Up genes represent genes highly expressed in pancreatic cancer patients with lymph node metastasis. Down genes represent genes highly expressed in pancreatic cancer patients without lymph node metastasis. **H** 0 gene was derived from the intersection between hub genes in green module and up genes in GSE10448. **I** 13 genes were derived from the intersection between hub genes in blue module and down genes in GSE10448

We performed GO enrichment and KEGG pathway analysis of the genes in green and blue modules, respectively. By GO enrichment, we found that the genes in green modules were significantly correlated with extracellular matrix organization, M-receptor interaction, extracellular matrix structural constituent, etc. (Fig. 2A); And the genes in blue modules were significantly correlated with chemical synaptic transmission, cell junction, calcium ion binding, etc. (Fig. 2B). By KEGG pathway analysis, we found that the genes in green modules were significantly correlated with ECM-receptor interaction, protein digestion and absorption, focal adhesion, PI3K-Akt signaling pathway and amoebiasis (Fig. 2C). The genes in blue modules were significantly correlated with insulin secretion, retrograde endocannabinoid signaling, neuroactive ligand-receptor interaction, nicotine addiction and GABAergic synapse (Fig. 2D).

To find hub genes in green and blue modules respectively, the correlation data of genes expression in each module was extracted via WGCNA and performed in Cytoscape via MCODE. According to the intensity of genes co-expression, gene groups with the highest score were selected, which represent hub genes in green and blue modules (Fig. 2E, F). In green module, COL5A1, FN1, THBS2, etc. were selected as hub genes (Fig. 2E), which might positively contribute to the lymph node metastasis. In blue module, FFAR1, SCG5, TMEM63C, etc. were selected as hub genes (Fig. 2F), which might negatively contribute to the lymph node metastasis.

### Further screening of the hub genes

To further select significant genes from hub genes, we obtained gene expression data of pancreatic tumors from GSE101448 (http://www.ncbi.nlm.nih.gov/geo/) and divided the patients into two groups according to whether exist lymph node metastasis. In volcano plot, there are many differentially expressed genes between two groups. Upregulated genes were enriched in patients with lymph node metastasis, while downregulated genes were highly expressed in patients without lymph node metastasis (Fig. 2G). Hub genes in TCGA and differential genes in GSE101448 were combined to analyze. There was no intersection of the hub genes in green module and upregulated genes in GSE101448 (Fig. 2H). For genes negatively related to lymph node metastasis, 13 genes were screened out between the hub genes in blue modules and the downregulated genes in GSE101448 (Fig. 2I).

**Fig. 3:**
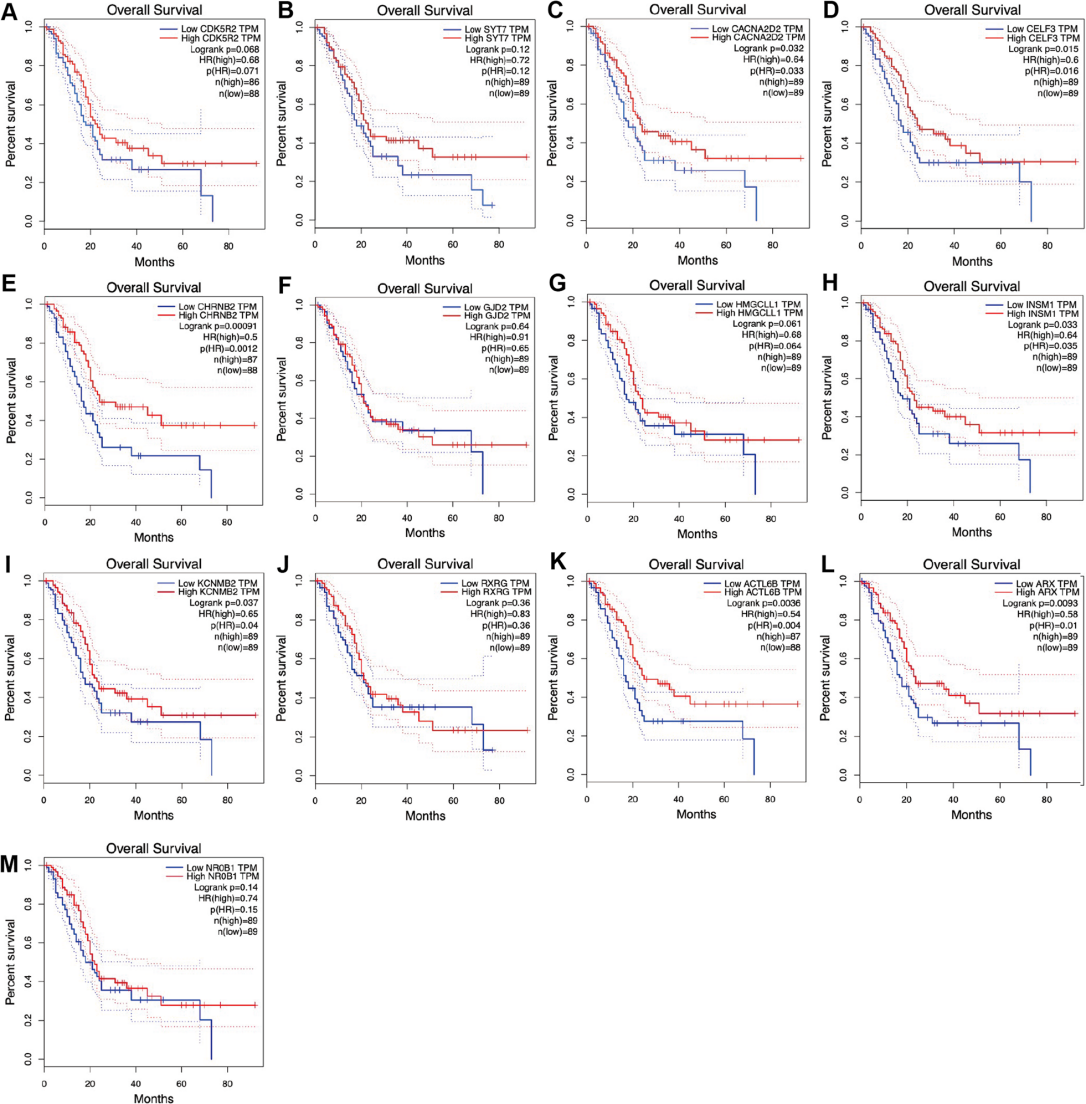
The overall survival among 13 genes in pancreatic cancer patients. **A**–**M** Majority of the selected genes are related to the longer survival of pancreatic cancer patients in the TCGA database. CHRNB2 showed the lowest hazard ratio (0.5) and P value (< 0.001) among the 13 genes

Considering key genes could influence lymph node metastasis and further affect prognosis, the relationship between the expression of these genes and the survival time of pancreatic cancer patients in TCGA database was further assessed. All of them showed negative relationship with overall survival time (Fig. 3). The expression levels of the 13 key genes in paired pancreatic cancer and paracancer tissue were further detected via GSE15471 (Additional file 1: Fig. S1). Most genes were expressed higher in paracancer tissue, which indicated their potential role in repressing cancer progression. Among them, the statistical significance between CHRNB2 and patient survival times was the most obvious (p = 0.0012) (Fig. 3E). Moreover, pancreatic cancer tissue expresses lower CHRNB2 than normal pancreas as well (Additional file 1: Fig.S1 E). Additionally, in the GO and KEGG analysis about blue module, CHRNB2 is the key gene participating in some significant pathways including “chemical synaptic transmission”, “potassium ion transmembrane transport”, “plasma membrane”, and “neuroactive ligand-receptor interaction”. Thus, the role of CHRNB2 in the lymph node metastasis of pancreatic cancer was further investigated.

### CHRNB2 attenuated migration and invasion ability of pancreatic cancer cell in an acetylcholine-independent manner

**Fig. 4:**
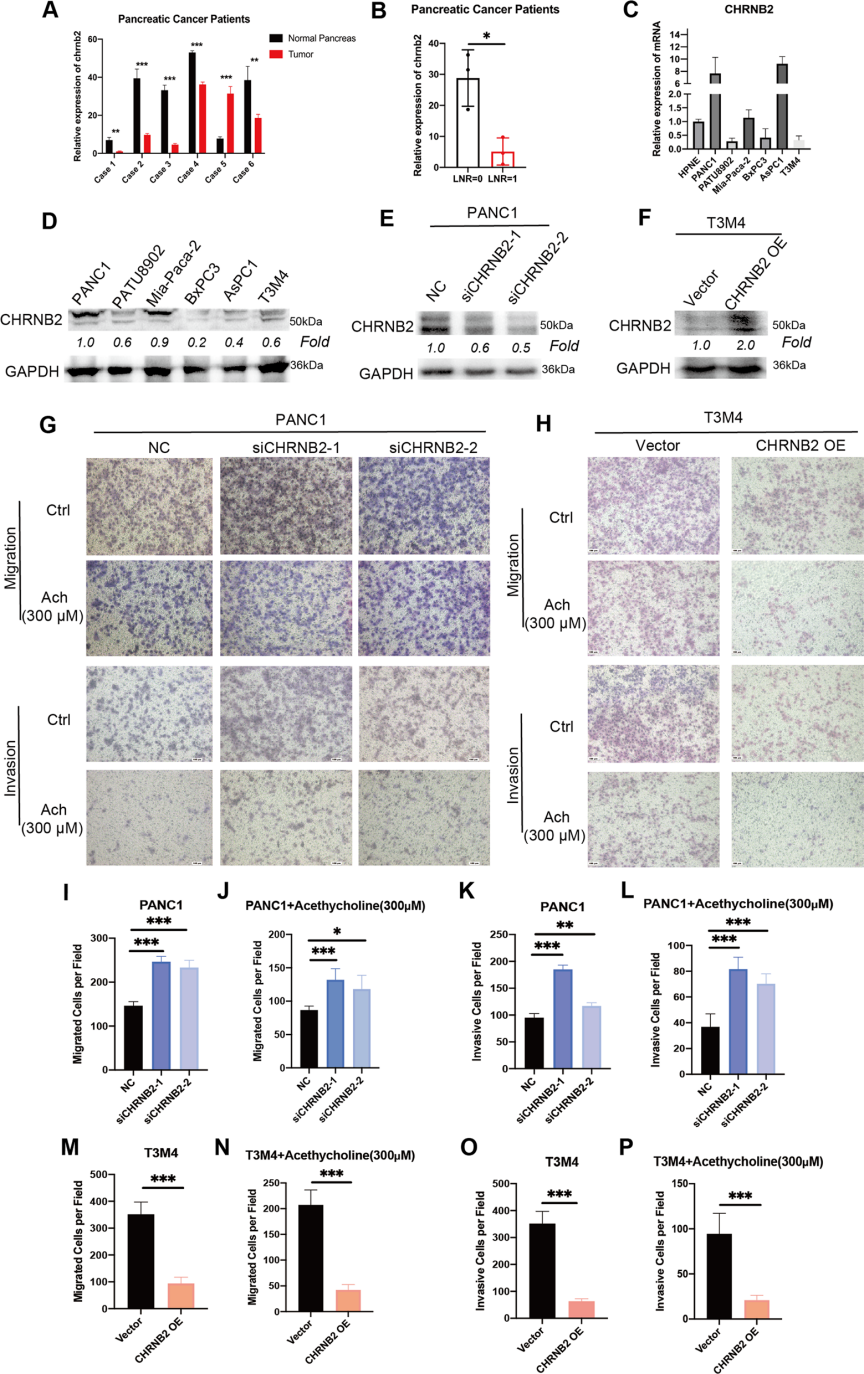
CHRNB2 represses the migration and invasion via ACh-independent mechanisms. **A** mRNA levels of CHRNB2 in 6 cases of pancreatic cancer with paired normal pancreas and tumor tissue. **B** CHRNB2 mRNA levels in the tumor tissue of 6 pancreatic cancer patients. 3 cases with LNR = 0, other 3 cases with LNR = 1, examined lymph node > 11. **C** mRNA levels of CHRNB2 in normal pancreatic ductal epithelial cell line and 6 pancreatic cancer cell lines. **D** Protein levels of CHRNB2 in 6 pancreatic cancer cell lines. **E** Efficiency of CHRNB2 knockdown by siRNA in PANC1. **F** Efficiency of CHRNB2 overexpression by plasmid in T3M4. **G**, **I**–**L** Downregulating CHRNB2 could ACh-independently increase the migration and invasion of PANC1. **H**, **M**–**P** Overexpressing CHRNB2 could ACh-independently decrease the migration and invasion of T3M4. (*: P < 0.05; **: P < 0.01; ***: P < 0.001)

To analyze the role of CHRNB2 in pancreatic cancer, baseline clinical variables between patients with high/low CHRNB2 expression in TCGA database were compared. Results suggested that patients with high CHRNB2 expression preferred to have lower T stages (T1 &T2) and LNR (Table 2), which were consistent with WGCNA results and the relationship between T stages and LNR. The expression of CHRNB2 was further compared between tumor and paired normal tissue in 6 pancreatic cancer patients. CHRNB2 was highly expressed in 5 patients (Fig. 4A). Moreover, High LNR patients (LNR = 1) expressed significantly lower CHRNB2 than patients with LNR = 0 (examined lymph node > 11) (Fig. 4B).

**Table 2:** Relationship between CHRNB2 expression and clinical information in pancreatic cancer patients

Variables	CHRNB2		Total	P value
	Low(n = 89)	High(n = 89)		
Sex				
Female	40	40	80	1
Male	49	49	98	
Age				
≤ 65	46	47	93	1
> 65	43	42	85	
T Stage				
T1&T2	7	24	31	0.002^**^
T2&T3	82	63	145	
LNR				
Low	36	50	86	0.0328^*^
High	50	36	86	
M stage				
M0	38	41	79	1
M1	2	3	5	
Histological grade				
G1&G2	61	65	126	0.328
G3&G4	26	24	50	
Tumor location				
Head	73	65	138	0.209
Non-head	16	24	40	
Somker				
No	32	33	65	0.65
Yes	43	36	79	
Alcohol history				
No	30	35	65	0.609
Yes	52	49	101	
Diabetes history				
No	54	54	108	0.928
Yes	20	18	38	
Chronic pancreatitis history				
No	61	67	128	0.085
Yes	10	3	13	
Family history of cancer				
No	25	22	47	0.474
Yes	28	35	63	

* P < 0.05

** P < 0.01

Considering the invasive ability of pancreatic cancer cells was significant in lymph node metastasis, the relationship between CHRNB2 and migration was explored. The mRNA and protein expression level of CHRNB2 in several pancreatic cancer cell lines (HPNE, PANC1, PATU8902, Mia-Paca-2, BxPC3, AsPC-1 and T3M4) were initially evaluated, among which, PANC1 cell line showed the highest expression level of CHRNB2 and T3M4 cell line showed the lowest expression level of CHRNB2 in both mRNA and protein levels (Fig. 4C, D). Therefore, PANC1 was selected for knockdown experiments, T3M4 was selected for overexpression experiments. The efficacy of siRNA and overexpression-plasmid were proved in protein level (Fig. 4E, F). Migration and invasion experiments were performed after knockdown and overexpression of CHRNB2. Since CHRNB2 encodes acetylcholine receptor protein, exogenous acetylcholine (ACh) (300uM) was added to find out if CHRNB2 functioned via Ach. Previous report has suggested that ACh could diminish the invasion of pancreatic cancer via repressing pERK signaling pathway [26]. Our results showed that downregulation of CHRNB2 significantly increased the migration and invasion ability of pancreatic cancer with or without ACh (300 μM) (Fig. 4G, I–L). Meanwhile, overexpression of CHRNB2 significantly weakened the migration and invasion ability of pancreatic cancer with or without ACh (300 μM) (Fig. 4H, M–P). These results indicated that CHRNB2 could attenuate migration and invasion ability of pancreatic cancer cells via Ach-independent mechanism.

### CHRNB2 inhibited the EMT process in pancreatic cancer

**Fig. 5:**
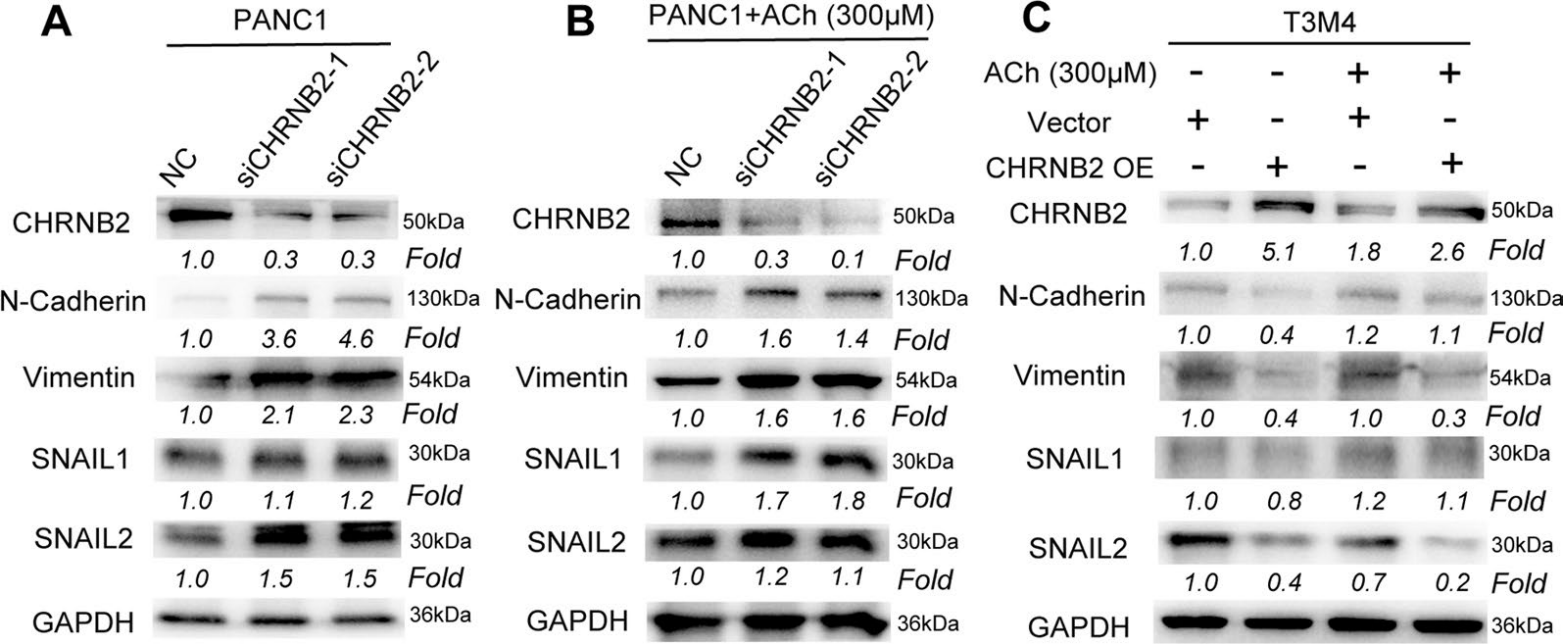
CHRNB2 represses EMT-related proteins via ACh-independent mechanisms. **A**, **B** Downregulating CHRNB2 could ACh-independently increase the expression of multiple EMT proteins including N-cadherin, Vimentin, SNAIL1, and SNAIL2 in PANC1. **C** Overexpressing CHRNB2 could ACh-independently decrease the expression of multiple EMT proteins including N-cadherin, Vimentin, SNAIL1, and SNAIL2 in T3M4

To further investigate the mechanism of decreased tumor migration and invasion caused by CHRNB2. We evaluated the relationship between CHRNB2 and Epithelial-to-mesenchymal transition (EMT) process, which is considered to foster the metastasis of malignant tumors, including pancreatic cancer [17, 27]. The expression of N-Cadherin, Vimentin, SNAIL1 and SNAIL2 were significantly upregulated after knockdown of CHRNB2 in ACh-independent mechanism (Fig. 5A, B). Correspondingly, the expression of N-cadherin, Vimentin, SNAIL1 and SNAIL2 were significantly downregulated after overexpression of CHRNB2 (Fig. 5C). Therefore, CHRNB2 was considered to inhibit the EMT process in pancreatic cancer cells.

### CHRNB2 inhibited Wnt/β-catenin pathway in pancreatic cancer

**Fig. 6:**
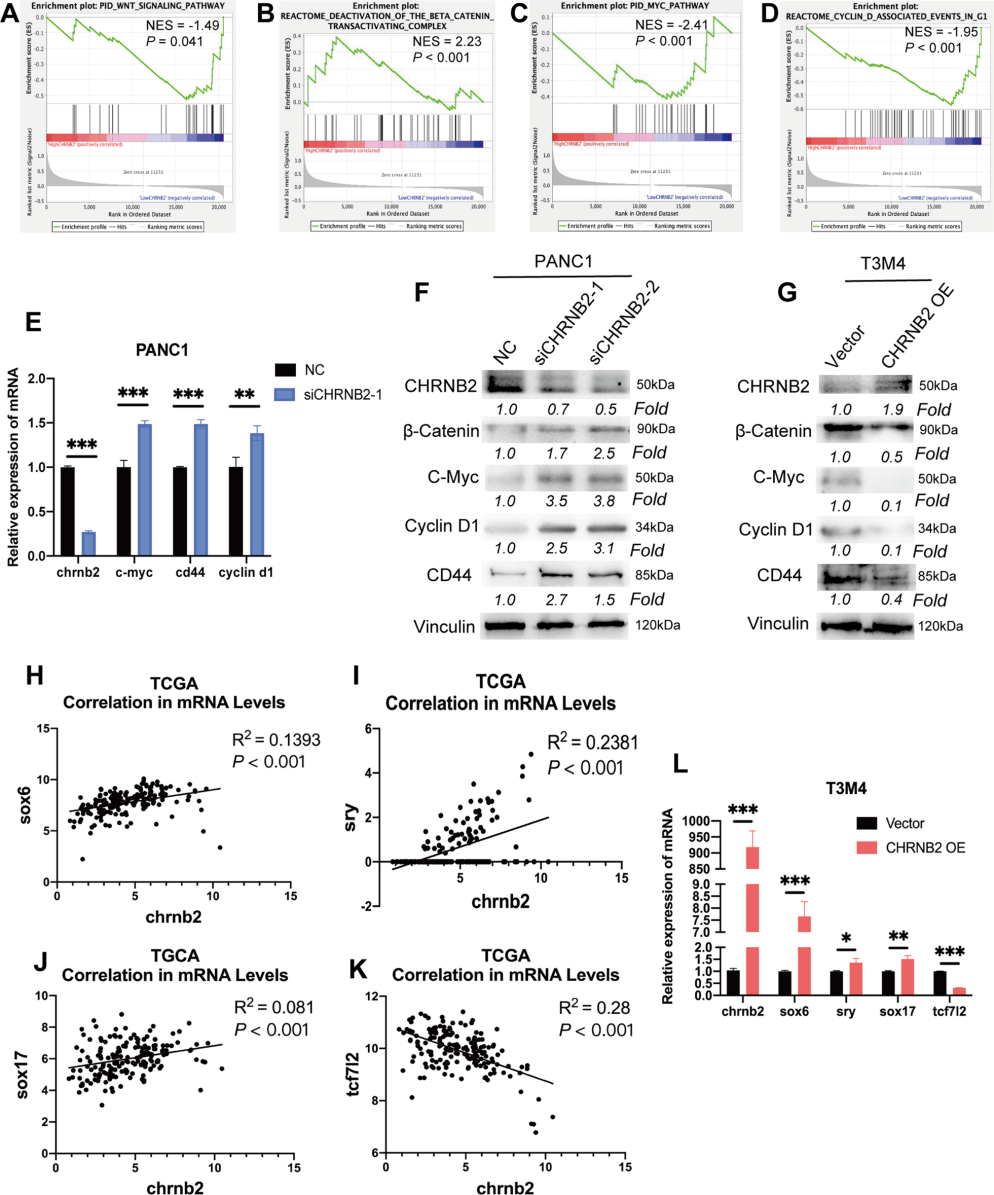
CHRNB2 negatively regulated β-catenin pathway. **A**–**D** GSEA results showed that CHRNB2 negatively related to the Wnt/β-catenin signaling and downstream MYC and Cyclin D1 pathways, but positively related to the deactivation pathway of β-catenin (NES: Normalized Enrichment Score). **E**, **F** Downregulating CHRNB2 could increase the expression of the β-catenin and its downstream proteins including C-Myc, Cyclin D1, and CD44 in both RNA and protein levels. **G** Overexpressing CHRNB2 could decrease the expression of the β-catenin and its downstream proteins including C-Myc, Cyclin D1, and CD44. **H**–**G** The correlation between CHRNB2 and the expression of SOX6, SRY, SOX17 and TCF7L2 in TCGA database. **L** Overexpressing CHRNB2 could enhance the expression of SOX6, SRY, SOX17, but repress the expression of TCF7L2 in T3M4. (*: P < 0.05; **: P < 0.01; ***: P < 0.001)

To explore the underlying mechanism of CHRNB2 that promote migration and invasion, TCGA database was employed and GSEA was performed to assess the relationship between CHRNB2 expression and various molecular pathways. The results indicated that CHRNB2 was negatively correlated with Wnt signaling pathway (NES = − 1.49, P = 0.041), MYC pathway (NES = − 2.41, P < 0.001) and Cyclin D1 associated pathway (NES = − 1.95, P < 0.001), but positively correlated with the deactivation of β-catenin pathway (NES = 2.23, P < 0.001) (Fig. 6A–D). Thus, CHRNB2 might negatively regulate the β-catenin pathway. Therefore, we accessed the expression level of downstream of β-catenin pathway. Since C-Myc, Cyclin D1 and CD44 are typical downstream of β-catenin pathway [28, 29]. The results illustrated that the expression of C-Myc, Cyclin D1 and CD44 were significantly improved after knockdown of CHRNB2 (Fig. 6E). In protein level, after CHRNB2 downregulation, the expression level of β-Catenin and its downstream proteins including C-Myc, Cyclin D1, and CD44 were increased (Fig. 6F). After CHRNB2 overexpression, the expression level of β-Catenin and its downstream proteins were increased (Fig. 6G). Considering CHRNB2 overexpression could repress Cyclin D1, which is an important regulator of cell cycle [30]. We detected the cell cycle alteration of pancreatic cancer cells after overexpressing CHRNB2. The results suggested that CHRNB2 could significantly prevent pancreatic cancer cells from entering S stage (Additional file 2: Fig. S2). In general, CHRNB2 might interfere β-catenin pathway and thus decreased the expression of downstream proteins of β-catenin, which further attenuated the migration and invasion ability of pancreatic cancer.

For the specific mechanism of CHRNB2 in downregulating β-catenin pathway, we utilized TCGA database and further detected the relationship between CHRNB2 expression and key genes which could regulate β-catenin pathway, such as SOX6 [30], SRY [31], SOX17 [32], and TCF7L2 [33]. Among them, SOX6, SRY, and SOX17 could repress β-catenin pathway, while TCF7L2 promote β-catenin pathway. The results suggested that CHRNB2 expression positively related to the expression of SOX6, SRY, and SOX17 (Fig. 6H–J), and negatively related to the expression of TCF7L2 (Fig. 6K). Moreover, the relationship between CHRNB2 and aforementioned β-catenin pathway regulators was further verified in pancreatic cancer cell lines after overexpressing CHRNB2 (Fig. 6L). Therefore, CHRNB2 might regulate β-catenin pathway through multiple and complex mechanisms.

### GSK-3 inhibitor rescued the inhibition of Wnt/β-catenin pathway by CHRNB2

**Fig. 7:**
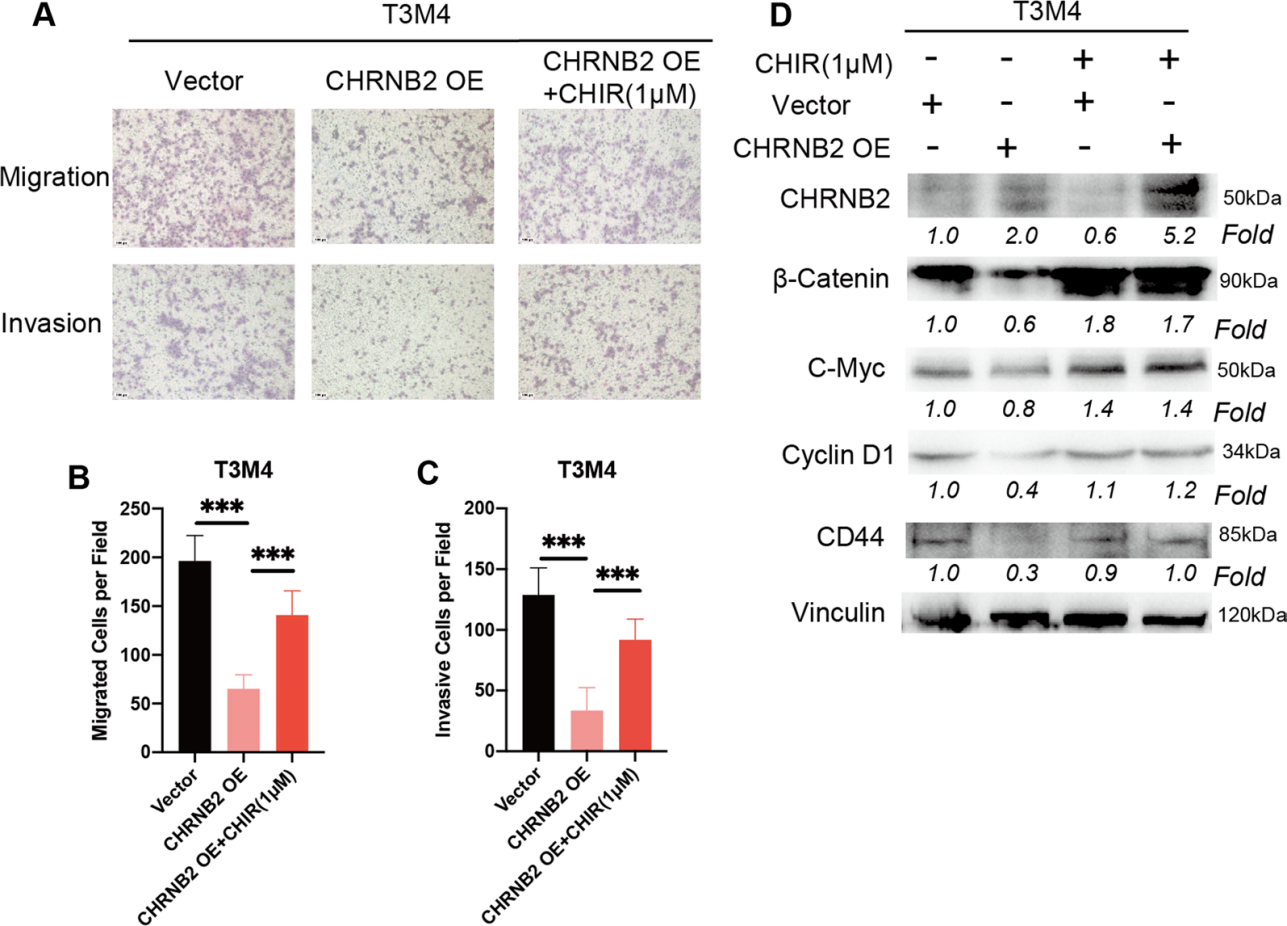
CHIR99021 could rescue the migration, invasion and β-catenin pathway after CHRNB2 overexpression. **A**, **B** In transwell migration and invasion assays, T3M4 were treated with DMSO as control or CHIR99021 (1 μM). Overexpressing CHRNB2 could decrease the migration and invasion of T3M4, CHIR99021 could obviously rescue the inhibition of CHRNB2. (D) After transfection, T3M4 were treated with DMSO as control or CHIR99021 (1 μM) for 48 h. CHIR99021 could rescue the effects of CHRNB2 overexpression and significantly improve the expression of β-catenin and its downstream proteins including C-Myc, Cyclin D1, and CD44. (CHIR: CHIR99021; ***: P < 0.001)

TO further certify the negative regulation between CHRNB2 and β-catenin pathway. Glycogen synthase kinase-3 (GSK-3) inhibitor, CHIR-99021, was employed to rescue the effects of CHRNB2 overexpression. GSK-3 functions as a part of the β-catenin destruction complex, which could induce the degradation of β-catenin [34]. Thus, CHIR-99021 was used to increase intracellular β-catenin levels and stimulate β-catenin pathway. Our results suggested that CHIR-99021 could reverse the inhibitory role of CHRNB2 in migration and invasion (Fig. 7A, B, C). In protein level, CHIR-99021 could significantly maintain the expression level of β-catenin and reverse the downregulated β-catenin pathway (C-Myc, Cyclin D1, CD44) caused by CHRNB2 overexpression (Fig. 7D). These results further strengthened the evidence that CHRNB2 attenuated cell migration and invasion through β-catenin pathway.

## Discussion

LNR is defined as the ratio between the number of positive lymph nodes and the total number of examined lymph nodes. Previous studies have identified LNR as a strong indicator for poor prognosis of patients with pancreatic cancer [35] and intraductal papillary mucinous neoplasms [36]. In the present study, we further confirmed that high LNR was associated with poor prognosis and tumor stage in patients with pancreatic cancer. There are two common but independent reasons that mediate lymph node metastasis, including the intrinsic migrative ability of cancer cells [37] and the lymphangiogenesis property of lymphatic endothelial cells [38]. To investigate the inner mechanism of lymph node metastasis, gene expression data of patients with pancreatic cancer from TCGA database was extracted and performed WGCNA algorithm in low and high LNR groups. In the present study, by using WGCNA, key modules associated with lymph node metastasis were obtained. And GO and KEGG analysis of the genes in these key modules were performed to find significant signaling pathways. The results showed that extracellular matrix played a role in lymph node metastasis, which might be induced by integrins and related ligands [39]. Chemical synaptic transmission and nervous related pathways were suggested to negatively regulate lymph node metastasis. However, there are still lack relative research. Further, hub genes in each module were identified through Cytoscape. After taking intersection with GSE101448, which contained clinical information about lymph node metastasis and RNA sequence data, 13 genes among hub genes were selected. Remarkably, we found CHRNB2 was negatively related with patient survival times with the most significant statistical difference.

In previous studies, there are few studies on the relationship between CHRNB2 and cancer. In gastric cancer, CHRNB2 could promote cancer progression by PI3K-AKT and JAK-STAT pathways with unclear mechanism [40]. However, our results indicate that CHRNB2 could repress EMT and metastasis in pancreatic cancer, which emphasized distinct molecular regulation among different cancer types. CHRNB2 is a subunit of nicotinic acetylcholine receptor [41], suggesting that its biological role might be induced by ACh. Recent research reported that Ach could inhibit pancreatic cancer progression by stimulating muscarinic receptors and suppressing pERK signaling [26]. Moreover, nicotinic acetylcholine receptors could transmit potassium and calcium as ion channels after binding with ACh. Previous study suggested that high potassium channel activity could inhibit the EMT of breast cancer via affecting β-catenin signaling [42]. Therefore, we supposed that ACh could promote the potassium channel activity of nicotinic acetylcholine receptor through binding with CHRNB2 and control pancreatic cancer metastasis. However, our results showed that CHRNB2 could repress the EMT of pancreatic cancer without ACh, suggesting CHRNB2 has ACh-independent functions.

EMT is considered to foster the metastasis of malignant tumors, including pancreatic cancer [17, 27]. During the process of EMT, the mesenchymal proteins are elevated, such as N-cadherin and Vimentin [43, 44]. Besides, the process of EMT is regulated by several transcription factors in pancreatic cancer, for example, SNAIL1 and SNAIL2 [45]. Wnt/β-catenin pathway plays an important role in EMT of various cancer types [46]. Wnt could bind with the receptors on the cell membrane and indirectly activate β-catenin. Additionally, the dislocation between E-cadherin and β-catenin also could promote its nuclear translocation and drive the expression of downstream EMT-related genes [47]. CHRNB2 has some potential ways to limit the activation of β-catenin and the expression of downstream genes. First, CHRNB2 is located on the cell membrane, which might directly tack β-catenin and inhibit its nuclear translocation. Second, considering CHRNB2 has significant positive relationship with the pathways deactivating β-catenin, it may directly activate some genes expression, such as SRY, SOX17 and SOX6, which could respectively inhibit the transcriptional ability of β-catenin [31], repress β-catenin expression [32], and promote the degradation of β-catenin [30]. Moreover, CHRNB2 negatively regulated TCF7L2, which could stabilize β-catenin and enhance the transcription of its downstream genes [33]. Thus, CHRNB2 might act β-catenin pathway via multiple mechanisms which deserve further study.

In conclusion, after bioinformational analysis and experimental verifications, we found that CHRNB2 could inhibit the migration and invasion of pancreatic cancer cells via limiting β-catenin pathway. It provides a possible strategy to repress the metastasis of pancreatic cancer, which may distinctly improve the overall survival of pancreatic cancer patients and deserve further study. For example, the mRNA of CHRNB2 could be loaded into engineered exosomes, which may increase the protein level of CHRNB2 in pancreatic cancer cells and prevent or alleviate metastasis [48]. Limitations still exist in our work. First, lymph node metastasis is dependent on both cancer cells and lymphatic endothelial cells. However, our research only focused on cancer cells. CHRNB2 might also play a role in restricting lymphoangiogenesis. Second, although the functions of CHRNB2 were confirmed in pancreatic cancer cells in vitro, animal experiments should also be performed which could further certify the role of CHRNB2 in vivo. Hence, further study is required to substantiate the correlation between CHRNB2 and lymph node metastasis in pancreatic cancer.

## Supplementary Information

**Additional file 1**: **Figure S1**. The expression conditions between tumor and paired normal pancreas among 13 genes in pancreatic cancer patients. **A**-**M** Compared to the paired normal pancreas, tumor tissue of most pancreatic cancer patients in GSE15471 has lower CHRNB2 expression. (**: P < 0.01; ***: P < 0.001).

**Additional file 2**: **Figure S2**. Flow cytometry showed that CHRNB2 arrested pancreatic cancer cells in the G1 phase. **A** Cell cycle flow chart of T3M4 with CHRNB2 overexpression. **B** Cell cycle histogram including G1, S, and G2 stages of T3M4 with CHRNB2 overexpression. (**: P < 0.01; ***: P < 0.001).

## Data Availability

Not applicable.
