# Supplementary figures and images for: CHRNB2 represses pancreatic cancer migration and invasion via inhibiting β-catenin pathway
Source: Cancer Cell Int. 2022 Nov 7;22:340. doi: 10.1186/s12935-022-02768-8 (PMC9641890; doi:10.1186/s12935-022-02768-8)

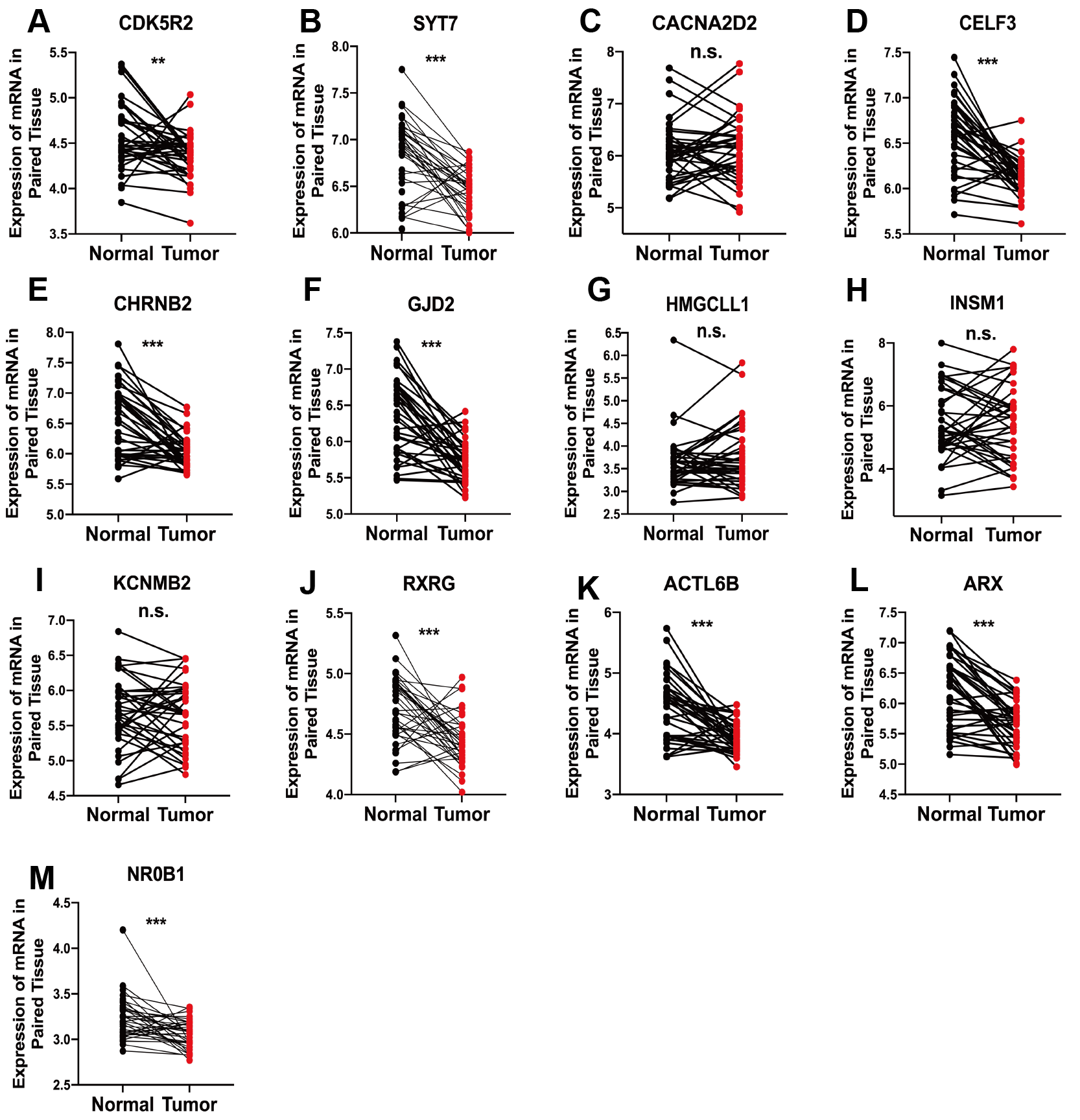

Supplement: Supplementary file 1 — Additional file 1: Figure S1. The expression conditions between tumor and paired normal pancreas among 13 genes in pancreatic cancer patients. A-M Compared to the paired normal pancreas, tumor tissue of most pancreatic cancer patients in GSE15471 has lower CHRNB2 expression. (**: P < 0.01; ***: P < 0.001). [file 12935_2022_2768_MOESM1_ESM.tif]

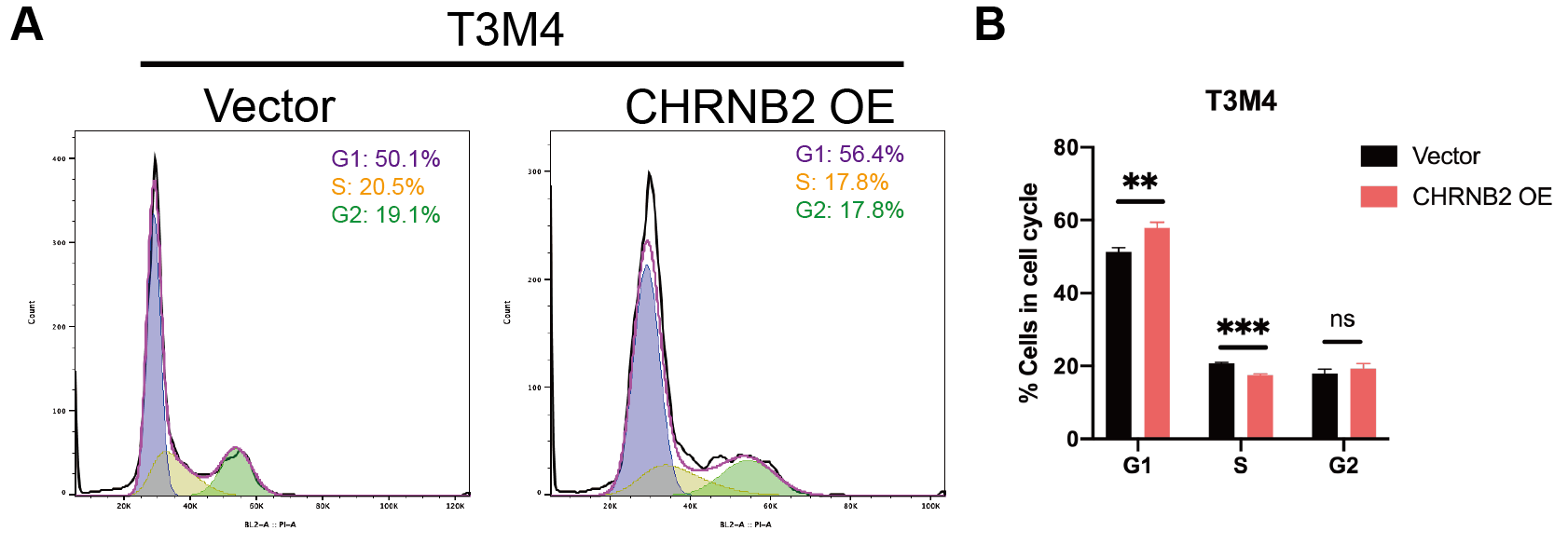

Supplement: Supplementary file 2 — Additional file 2: Figure S2. Flow cytometry showed that CHRNB2 arrested pancreatic cancer cells in the G1 phase. A Cell cycle flow chart of T3M4 with CHRNB2 overexpression. B Cell cycle histogram including G1, S, and G2 stages of T3M4 with CHRNB2 overexpression. (**: P < 0.01; ***: P < 0.001). [file 12935_2022_2768_MOESM2_ESM.tif]
